# The Role of Hsp90 in Retinal Proteostasis and Disease

**DOI:** 10.3390/biom12070978

**Published:** 2022-07-12

**Authors:** Kalliopi Ziaka, Jacqueline van der Spuy

**Affiliations:** UCL Institute of Ophthalmology, 11-43 Bath Street, London EC1V 9EL, UK; kalliopi.ziaka.16@ucl.ac.uk

**Keywords:** chaperone, co-chaperone, heat shock protein, Hsp90, inherited retinal disease, photoreceptor, phototransduction, proteostasis

## Abstract

Photoreceptors are sensitive neuronal cells with great metabolic demands, as they are responsible for carrying out visual phototransduction, a complex and multistep process that requires the exquisite coordination of a large number of signalling protein components. Therefore, the viability of photoreceptors relies on mechanisms that ensure a well-balanced and functional proteome that maintains the protein homeostasis, or proteostasis, of the cell. This review explores how the different isoforms of Hsp90, including the cytosolic Hsp90α/β, the mitochondrial TRAP1, and the ER-specific GRP94, are involved in the different proteostatic mechanisms of photoreceptors, and elaborates on Hsp90 function when retinal homeostasis is disturbed. In addition, several studies have shown that chemical manipulation of Hsp90 has significant consequences, both in healthy and degenerating retinae, and this can be partially attributed to the fact that Hsp90 interacts with important photoreceptor-associated client proteins. Here, the interaction of Hsp90 with the retina-specific client proteins PDE6 and GRK1 will be further discussed, providing additional insights for the role of Hsp90 in retinal disease.

## 1. Phototransduction and Protein Folding in Photoreceptors (PR)

Photoreceptor cells are highly specialized sensory neurons in the retina, and are essential for converting light into a neural signal, a fundamental process which initiates vision. In the mammalian retina, there are two types of photoreceptor cells, the rods and the cones. Both rods and cones are adjacent to the retinal pigment epithelium (RPE), a monolayer of pigmented cells which is vital for the normal function and survival of photoreceptors [1]. Morphologically, photoreceptors consist of a synaptic terminal, a nuclear region, and an inner segment (IS) and outer segment (OS) which are connected by a connecting cilium (CC). The OS of both cell types consists of closely spaced membranous discs containing photopigment molecules, called opsins, which are coupled to a light-absorbing chromophore (retinal, an aldehyde of vitamin A). Opsins are responsible for tuning the absorption of light to a specific wavelength of the light spectrum. The rod OS contains the rod-specific photopigment rhodopsin, whereas the cone OS contains one of the three cone-opsins, S-opsin, M-opsin, or L-opsin. Rhodopsin, with a peak absorption (*λ*_max_) of ~500 nm, functions during dim light conditions allowing scotopic vision, whereas cone opsins are responsible for processing wavelengths ranging from ~350 to 560 nm, thus allowing colour vision [2]. Rods and cones share the same cellular mechanism of light detection, a process known as phototransduction.

Phototransduction is a complex mechanism in which light is converted into an electrical signal through the sequential activation of signalling proteins. In rods, phototransduction is activated by the photoisomerization of the rhodopsin-bound chromophore 11-*cis*-retinal to all-*trans* retinal, inducing a conformational change in rhodopsin to its activated form metarhodopsin II. Metarhodopsin II stimulates the trimeric G-protein transducin by catalysing the exchange of GDP for GTP on the α-subunit. The GTP-associated α-subunit of transducin dissociates from the β and γ subunits and activates PDE6, a phosphodiesterase that hydrolyses cGMP. The decreased concentration of cGMP in the OS results in the closure of cGMP-gated channels in the plasma membrane, and the cessation of sodium and calcium influx, which, in turn, leads to the hyperpolarisation of the rod cell and the inhibition of glutamate release at the synaptic terminal [3]. A series of biochemical reactions is required for photoreceptors to return to their inactive state. This involves another network of proteins which restore the various activated components to their inactive state. G-protein-coupled receptor kinase 1 (GRK1) phosphorylates metarhodopsin II, inducing a conformational change that enables the binding of arrestin, leading to its inactivation. PDE6 is inactivated upon GTP hydrolysis of the transducin α-subunit, a process that is facilitated by the GTPase-activating protein (GAP) complex, consisting of RGS9 (regulator of G-protein-signalling isoform 9) and G-protein β-subunit or G-protein β-subunit-like protein [4]. As a result, free cGMP concentration returns to normal levels (depolarised state) due to the activation of guanylyl cyclase activating proteins (GCAP) and the cGMP-synthesizing enzyme guanylate cyclase (GC). The phototransduction cascade in cone photoreceptors is similar to that in rods and is mediated by homologous phototransduction proteins [5,6]. However, while rods generate a detectable single photon response for maximal sensitivity in dim light conditions, cones are less sensitive than rods and require the simultaneous activation of tens to hundreds of opsin molecules in bright light conditions to generate a detectable response. The high spatial and temporal resolution of cone-mediated vision is made possible by the rapid kinetics of activation and inactivation, the trade-off of which is low amplification and sensitivity. In contrast, the trade-off of the high amplification gain of rods in dim light is their slow kinetics [5,6]. The adaption of rods and cones that shift their dynamic range towards dim and bright light detection respectively places a great metabolic demand on photoreceptors, as the visual cycle requires high amounts of energy for the phototransduction components to coordinate and function together. Moreover, constant triggering of phototransduction causes photooxidative stress to the OS components which need to be constantly replaced to avoid permanent damage. This is achieved by synthesis of new OS disks at the base of the OS and shedding of the OS tips which are phagocytosed by the RPE [7]. Interestingly, RPE cells have the highest phagocytic activity in the body, highlighting the intense metabolic demands of OS renewal [8,9]. To maintain this ability while performing their normal biological function, photoreceptors rely on high levels of protein synthesis, and the correct folding, assembly, trafficking, and degradation of various protein components. High levels of protein synthesis of the phototransduction components occur in the IS which must be continuously translocated through the connecting cilium to their site of action in the OS. The balance of these processes in the photoreceptor cell is called protein homeostasis or “proteostasis”.

## 2. The Importance of Hsp90 Isoforms in Retinal Proteostasis

Proteostasis is maintained and controlled by an extensive network of molecular chaperones, proteolytic systems, and their regulators, termed the proteostasis network (PN). To ensure the correct folding and degradation of misfolded proteins, the PN includes sophisticated protein quality control (PQC) mechanisms, of which the chaperone Hsp90 is a vital component. Hsp90 is an ATP-dependent protein that is universally found in various cellular compartments, such as the cytosol, the ER, and the mitochondria. There are five Hsp90 members, which according to published guidelines for HSP nomenclature are categorised under the HSPC family and include the cytosolic HSPC1 (HSP90AA1), HSPC2 (HSP90AA2), HSPC3 (HSP90AB1), the endoplasmic reticulum (ER) HSPC4 (GRP94), and the mitochondrial HSPC5 (TRAP1) isoforms [10]. These isoforms participate in different PQC systems in the various compartments of the cell with a common aim to support the folding or refolding and stability of client proteins. Hsp90 functions as a dimer, with each protomer within the Hsp90 dimer comprising an N-terminal ATP-binding domain, a middle domain, and a constitutively dimerised C-terminal domain. Hsp90 chaperone activity is coupled with ATP hydrolysis, wherein ATP binds to an open conformation of Hsp90, which induces the transient dimerisation of the N-terminal domains and ATP hydrolysis with subsequent release of the client protein (reviewed by [11,12]). The Hsp90 cycle facilitates the folding, maturation, or assembly of near-native client proteins, of which there are several hundred (https://www.picard.ch/downloads/Hsp90interactors.pdf accessed on 3 July 2022). Hsp90 co-chaperones interact non-covalently with Hsp90 to modulate the Hsp90 cycle or specifically target client proteins to Hsp90 (reviewed by [11,12]).

### 2.1. Cytosolic Hsp90

**Figure 1:**
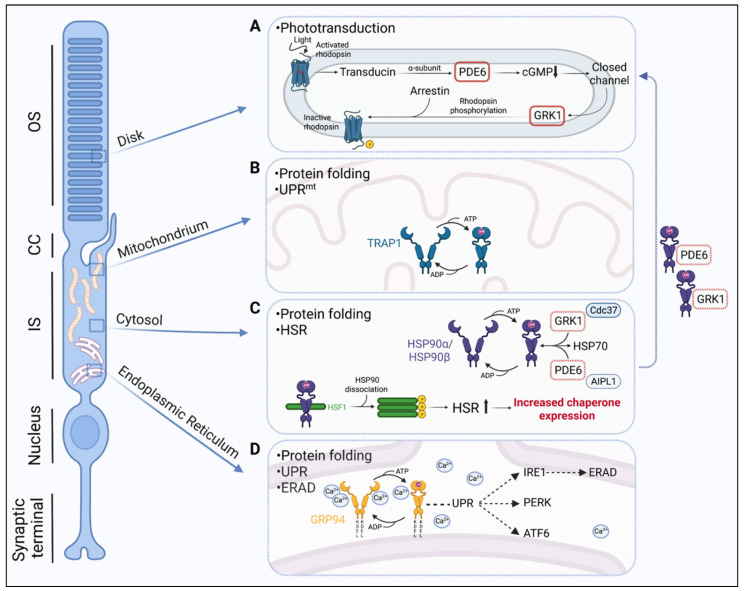
The roles of Hsp90 in photoreceptor proteostasis. (**A**) PDE6 and GRK1 are important components of phototransduction activation and deactivation, respectively. PDE6 and GRK1 are synthesised in the photoreceptor inner segment and translocated to the outer segment via the connecting cilium. Both PDE6 and GRK1 are Hsp90 client proteins. (**B**) The mitochondrial isoform TRAP1 is involved in protein folding and the mitochondrial unfolded protein response (UPR^mt^). (**C**) The cytosolic isoforms Hsp90α/Hsp90β participate in protein folding in association with the Hsp70 folding machinery, and as a part of the heat-shock response (HSR) by regulating the activation of heat-shock transcription factor 1 (HSF1). During stress, Hsp90 together with other chaperones dissociate from HSF1, which then trimerizes and is activated via phosphorylation. Activated HSF1 translocates to the nucleus and stimulates the expression of molecular chaperones. (**D**) ER-associated GRP94 is important in protein folding and ER protein quality control mechanisms, the UPR, and ER-associated degradation (ERAD). The UPR involves three signalling pathways mediated via ER transmembrane protein folding sensors, the inositol-requiring protein-1 (IRE1), the protein kinase RNA (PKR)-like ER kinase (PERK), and the activating transcription factor 6 (ATF6). Activation of the UPR branches leads to the increased expression of proteins, such as molecular chaperones, folding catalysts, subunits of the translocation machinery (Sec61 complex), ERAD molecules, and antioxidants. Created with BioRender.com.

The cytosolic isoforms of Hsp90 participate in protein folding as a part of the heat shock response (HSR) and the Hsp90/Hsp70 protein folding machinery (Figure 1). The HSR is an orchestrated process that leads to the rapid transcription of selective genes encoding cytosolic molecular chaperones, also known as heat shock proteins (HSPs). The transcriptional activation of HSPs is regulated by transcription factors known as heat shock factors (HSFs) [13]. HSF1 is the key regulator of the HSR leading to HSP induction in response to stress. In the absence of stress, monomeric HSF1 is maintained in an inactive state by interaction with molecular chaperones in the cytosol, including Hsp90 [14]. In the presence of stress, HSF1 is converted from an inactive monomer to an active DNA-binding trimer, and this trimerization process involves the dissociation of Hsp90 and co-chaperones from its regulatory domain [15]. The trimerized HSF1 translocates to the nucleus and binds to heat shock elements (HSE) in the promoters of target genes that promote the transcription of Hsp90 and other HSPs [16].

In addition to its role in the HSR, cytosolic Hsp90 also functions as part of the Hsp90/Hsp70 protein folding machinery, in which Hsp90 targets client proteins, early folding intermediates in a near native state, and in concert with Hsp70, facilitates the thermodynamically favourable maturation of these clients [17]. In mammalian cells, including photoreceptors, there are two major cytosolic Hsp90 isoforms, the stress-inducible Hsp90α (HSPC1) and the constitutively expressed Hsp90β (HSPC3), which share 85% sequence identity [18] (Figure 1). The less abundant Hsp90α A2 (HSPC2) isoform is identical to Hsp90α with the exception of an N-terminal extension in Hsp90α. A recent study showed that Hsp90α deficiency in mice can cause rhodopsin retention in the IS and eventually lead to retinal degeneration. Further investigation revealed that microtubule-associated protein 1B (MAP1B), which is important for microtubule stabilization, was associated with Hsp90α and significantly reduced in Hsp90-deficient mice by proteasomal degradation. The authors suggested that Golgi organisation and vesicle transportation, which both rely on stable microtubules, are disrupted and this could be the underlying cause of photoreceptor degeneration [19].

### 2.2. ER-Associated GRP94

The ER has its own network of molecular chaperones which ensure that correctly folded proteins are produced and exit from the organelle for further processing. The glucose-regulated protein 94 (GRP94) (HSPC4) is a key regulator of the ER quality control mechanism and its residence in the ER is facilitated by its distinct C-terminal sequence KDEL, which serves as an ER retrieval signal for the KDEL receptor [20] (Figure 1). GRP94, together with BiP (GRP78), are two of the most abundant proteins in the ER [21] and play a significant role in regulating the ER unfolded protein response (UPR). The UPR involves the activation of a well synchronised set of signalling pathways directed by ER-resident transmembrane proteins that include inositol-requiring protein-1 (IRE1), the protein kinase RNA (PKR)-like ER kinase (PERK), and the activating transcription factor 6 (ATF6) [22] (Figure 1). During stress overload, the UPR branches respond by stimulating the expression of UPR-targeted genes which encode proteins, such as molecular chaperones, folding catalysts, subunits of the translocation machinery (Sec61 complex), ER-associated degradation (ERAD) molecules, and antioxidants. This activation leads to the upregulation of the protein folding and degradation capacity and the inhibition of protein synthesis in order to alleviate the stress and restore the equilibrium in the ER. Specifically in photoreceptors, GRP94 has been shown to be involved in opsin quality control as it forms a complex with mutant opsins and other chaperones (BiP) [23]. Apart from protein folding, ER quality control involves ERAD, a mechanism to detect misfolded proteins and tag them for proteasomal degradation (Figure 1). Evidence from Christianson et al. (2008) [24] showed that GRP94 actively participates in ERAD, when α1-antitrypsin, an ERAD substrate, failed to degrade in GRP94-depleted cells. Misfolded rhodopsin is also subjected to ERAD [25,26], and it has been suggested that GRP94 and BiP might be involved in the recognition of the non-glycosylated ER-retained misfolded opsins [26]. Another important feature of GRP94 is its ability to bind calcium and maintain calcium homeostasis in the ER. The ER quality control machinery is coupled to the storage and utilization of calcium [27]. Most calcium in the ER is stored bound to proteins and GRP94 is one of the most important calcium-binding proteins [20]. A reduction in total calcium levels can strongly affect protein folding and change the molecular chaperone selection in the ER [28,29,30].

### 2.3. Mitochondrial TRAP1

The tumour necrosis factor receptor associated protein 1 (TRAP1) is the mitochondria-specific Hsp90 isoform (HSPC5) and has distinct structural and functional properties (Figure 1). Structurally, TRAP1 is similar to the cytosolic Hsp90 isoforms, with the exception of a cleavable N-terminal mitochondrial localization signal and an N-terminal extension or ‘strap’ that provides stability in its ‘closed’ conformation [31]. Functionally, TRAP1 participates in the maintenance of mitochondrial integrity, protein folding and response to proteotoxic stress in mitochondria, and protection from oxidative stress damage [31,32]. Similarly to the ER, mitochondria are particularly vulnerable to the disturbance of proteostasis due to their high intrinsic protein folding demands. Hence, they have developed their own protective mechanism to overcome proteotoxic stress, known as the mitochondrial unfolded protein response (UPR^mt^) [33]. Similar to the HSR and ER-specific UPR, UPR^mt^ is a transient transcriptional change in response to proteotoxic stress that is regulated by mitochondria. It was first described by Martinus et al. (1996) [34] as the transcriptional activation of mitochondria-specific chaperones and depends on mitochondrial-nuclear communication. The activation of UPR^mt^ promotes protein folding, limits protein import, and reduces the translation of mitochondrial proteins. The UPR^mt^ has been most extensively studied in *C. elegans* and there is only a small number of studies that have attempted to characterise it in mammalian cells [34,35,36]. Similarly to the ER-specific UPR, studies have shown that UPR^mt^ elicits a multi-axis response that is regulated by several proteins and leads to distinct molecular outcomes (reviewed by [37]). However, the exact molecular mechanisms and regulators of the different UPR^mt^ pathways remain largely unexplored, especially in disease. A study in *Drosophila* showed that modulation of TRAP1 expression led to the nuclear translocation of the transcription factor Dve which induced the expression of the mitochondrial chaperonin Hsp60, mitochondrial Hsp70, and a putative protease, CG5045, suggesting that TRAP1 is able to activate the UPR^mt^. The same study found that TRAP1 modulation could significantly improve health span, potentially by activation of the UPR^mt^ [38]. In recent years, a functional interaction between ER and mitochondria during stress has been the focus of scientific interest. TRAP1 has been shown to have an important role in this ER–mitochondria interaction since it can potentially regulate the ER-associated UPR [39]. In photoreceptors, the elongated mitochondria extend almost the entire length of the IS, and are critical in meeting the high energy demands of photoreceptors for protein synthesis and phototransduction in the OS as well as serving as a calcium store. The photoreceptor TRAP1 is therefore likely to play a critical role in the maintenance of photoreceptor homeostasis and the UPR^mt^ (Figure 1).

## 3. The Role of Hsp90 in Retinal Disease

### 3.1. Hsp90 and the Stress Response in Retinal Disease

Evidently, Hsp90 is of high importance in the retina because of the many vital roles it has in the different PQC mechanisms of proteostasis. Its importance in retinal homeostasis can be further highlighted by exploring its role in retinal disease paradigms. Currently, 280 genes (316 genes and loci) are associated with inherited retinal degeneration (IRD) (https://sph.uth.edu/retnet/home.htm, accessed on 3 July 2022). The inheritance of IRDs can be autosomal recessive, autosomal dominant, or X-linked, and IRDs can furthermore be progressive or stationary, as well as non-syndromic or part of a wider syndrome. Non-syndromic retinal dystrophies are further classified as macular dystrophies, cone and cone–rod dystrophies, rod–cone dystrophies, or chorioretinopathies [40]. Retinitis pigmentosa (RP) describes a group of retinal degenerative rod–cone dystrophies that are primarily characterized by the loss of rod photoreceptors, as well as the subsequent degeneration of cones. Mutations in rhodopsin are the most common cause of autosomal dominant retinitis pigmentosa (adRP) [2]. The photoreceptor stress machinery has been found to be induced in various models of rhodopsin misfolding. For example, the upregulation of the UPR and of the HSR, including increased levels of Hsp90 and Hsp70, has been observed in the P23H-1 transgenic rat, in which mutant rhodopsin Pro23His (P23H) is misfolded and retained in the ER [41]. Upon treatment with arimoclomol, an HSR co-inducer, Hsp90 and Hsp70 levels were further elevated, and this was associated with decreased rhodopsin aggregation, photoreceptor rescue, and improved visual responses [41]. The role of Hsp90 in the upregulation of the UPR and HSR is likely to be important in other retinal diseases caused by protein misfolding, in which the maintenance and regulation of the vast array of structural and functional proteins is critical for normal photoreceptor homeostasis.

### 3.2. Hsp90 Inhibition in Retinal Disease

Additional evidence of the significance of Hsp90 in retinal dystrophies, including RP, arise from the consequences of its pharmacological inhibition. Hsp90 inhibition can elicit a dual effect, leading to the proteasome-mediated degradation of its client proteins or the disruption of the chaperone complex with HSF1 and the activation of the HSR, leading to the upregulation of molecular chaperones. Therefore, the potential of Hsp90 inhibitors to manipulate the photoreceptor stress machinery has been explored in various studies. A list of Hsp90 inhibitors that have been used in models of retinal disease is summarized in Table 1. The Hsp90 inhibitor 17-*N*-allylamino-17-demethoxygeldanamycin (17-AAG), also known as tanepsimycin, has been shown to protect against rhodopsin aggregation and toxicity in a cell model of P23H rhodopsin [42]. Two other Hsp90 inhibitors, geldanamycin (GA) and radicicol, also showed a similar effect on alleviating the toxic gain-of-function mechanisms of P23H rhodopsin in vitro, although this effect was less potent compared to 17-AAG [42]. The amelioration of P23H rhodopsin aggregation was not observed in mouse embryonic fibroblasts from HSF-1 knock-out mice, suggesting that the protection depends on HSF1 and the activation of the HSR [43]. In accordance with these findings, the systemic administration of 2-amino-7,8-dihydro-6H-pyrido[4,3-d]pyrimidin-5-one NVPHSP990 (HSP990), a blood brain barrier permeable Hsp90 inhibitor, activated HSF-1 and induced the upregulation of molecular chaperones in the retina of P23H transgenic rats [43]. This HSP990-mediated stimulation of the stress machinery was associated with reduced rhodopsin aggregation and mislocalisation, improved visual function, and photoreceptor survival several weeks after a single drug dose [43]. Hsp90 inhibition has also been reported to be protective in another form of adRP, RP10, which is caused by mutations in the inosine 5′-monophosphate dehydrogenase type 1 (IMPDH1) gene [44]. Systemic delivery of 17-AAG, facilitated by the RNA interference-mediated modulation of the inner blood–retina barrier, protected against photoreceptor degeneration in the Arg224Pro (R224P) mutant IMPDH mouse model, by promoting the expression of HSPs, including Hsp90, which, in turn, reduced the formation of IMPDH aggregates [45]. These studies highlight the potential neuroprotective effects of Hsp90 inhibition in retinal protein misfolding disorders via upregulation of the HSR.

It has also been shown, however, that prolonged inhibition of Hsp90 in the retina may also play a detrimental role in photoreceptor proteostasis as a consequence of the degradation of key Hsp90 client proteins. The rhodopsin mutant Arg135Leu (R135L) is hyperphosphorylated and constitutively bound to arrestin, thereby disrupting vesicular traffic in photoreceptors. 17-AAG enhanced the vectorial transport of R135L rhodopsin to the OS by suppressing the endocytosis defect that characterises this mutation, thereby restoring R135L rhodopsin localization to the WT phenotype in rat retinae [43]. In an in vitro cell model of R135L rhodopsin, Hsp90 inhibition by 17-AAG similarly blocked the recruitment of arrestin to R135L rhodopsin and led to a reduction in the aberrant endocytosis of R135L rhodopsin [43]. Interestingly, this effect was HSF1-independent as 17-AAG rescued the intracellular accumulation of R135L rhodopsin and restored the cytosolic localization of arrestin in HSF-1 knock-out mouse embryonic fibroblasts [43]. It was hypothesized that Hsp90 inhibition may instead mediate its effect on R135L rhodopsin by client-mediated degradation. Indeed, prolonged Hsp90 inhibition with HSP990 in vivo led to a post-translational reduction in GRK1 and PDE6 protein levels, identifying them as Hsp90 clients. Hsp90 inhibition in cells led to the rapid proteasomal degradation of newly synthesised GRK1 confirming a requirement for Hsp90 for GRK1 maturation and function. The effect of Hsp90 inhibition on R135L rhodopsin was therefore attributed to the fact that GRK1 was identified as an Hsp90 client protein, and Hsp90 inhibition decreased GRK1 levels resulting in reduced R135L phosphorylation and subsequently, reduced arrestin binding.

### 3.3. Ocular Toxicities in Clinical Trials of Hsp90 Inhibition

These findings have important implications for the pharmacological manipulation of molecular chaperones as a therapeutic approach for retinal disease. Despite the plethora of evidence that Hsp90 inhibition can provide protection in the diseased retina as described above, reports from clinical trials in oncology highlight ocular toxicities that have emerged as an important clinical concern (Table 2). Hsp90 N-terminal inhibitors, including ansamycin derivatives (17-dimethylaminoethylamino-17-demethoxygeldanamycin (17-DMAG)), resorcinol derivatives (AT13387, AUY922), and benzamide derivatives (SNX-5422 (PF-04929113)), have been associated with visual disturbances, such as blurred vision, photopsia, night blindness, photophobia, and retinopathy [47,48,49,50,51,52,53,54,55,56,57]. In addition, some preclinical studies have reported that severe retinal degeneration occurred in rats and beagle dogs after treatment with Hsp90 inhibitors [46,58,59].The oral administration of the Hsp90 scaffold N-terminal inhibitor CH5164840 led to a loss of pupillary light reflex, abnormal electroretinographic (ERG) responses, and histological changes in the photoreceptor outer nuclear layer, including photoreceptor degeneration, in beagle dogs [58]. Similarly, intravenous administration of 17-DMAG or AUY922 promoted photoreceptor cell death in Sprague Dawley (SD) rats in addition to the upregulation of the HSR [46], and AUY92-induced abnormal ERG responses, and photoreceptor OS disorganization in Brown Norway and Wistar rats [59]. However, while ocular effects have been widely reported in preclinical and clinical studies of certain Hsp90 inhibitors, visual disturbances have not been reported for all Hsp90 inhibitors, including 17-AAG and the resorcinol derivative ganetespid. Preclinical studies comparing the ocular toxicity of 17-DMAG, 17-AAG, AUY922, and ganetespid suggest that the extent of ocular toxicity correlates with the retinal biocompatibility and clearance rate of the compound, with high levels of accumulation and prolonged inhibition of Hsp90 in the retina, leading to photoreceptor cell death [46]. Therefore, the inhibition of Hsp90 as a therapeutic approach in the retina is clearly a double-edged sword, whereby Hsp90 inhibition can both induce a neuroprotective response but also lead to ocular toxicity upon prolonged retinal accumulation. The mechanism of retinal toxicity as a consequence of Hsp90 inhibition is poorly understood; however, a possible explanation is that the ocular toxicity observed upon prolonged Hsp90 inhibition might be mediated by the disruption caused to important Hsp90 client proteins in the retina. As described previously, GRK1 biosynthesis requires Hsp90, and prolonged Hsp90 inhibition via systemic administration of HSP990 reduced GRK1 and PDE6 levels post-translationally, suggesting that the Hsp90 client list includes important components of the phototransduction cascade. More recently, Transient Receptor Potential cation channel subfamily M member 1 (TRPM1) has been identified as another potential Hsp90 client protein in the retina [57]. TRPM1 is a constitutively open calcium entry channel primarily expressed in skin melanocytes and retinal ON-bipolar cells in the inner nuclear layer. The treatment of mice with AUY922 resulted in increased apoptosis in the photoreceptor outer nuclear layer, disorganization of the photoreceptor outer segments, disruption of RPE cells, and a dose-dependent decrease in TRPM1 via disruption of the interaction with Hsp90 [57].

## 4. Hsp90 Client Proteins in the Retina

Whilst PDE6, GRK1, and TRPM1 have been identified as Hsp90 client proteins in the retina, only PDE6 and GRK1 are specifically expressed in photoreceptor cells and are important components of the phototransduction cascade. An in-depth understanding of the mechanisms underlying the specific recruitment of PDE6 and GRK1 to Hsp90 is crucial to understand the biogenesis of these important phototransduction proteins, not only in the healthy retina but also in retinal diseases associated with these Hsp90 clients, and the review will henceforth focus on mechanistic and structural insights into PDE6 and GRK1 as Hsp90 client proteins. Whilst the Hsp90-PDE6 chaperone complex has been investigated in depth, less is known regarding GRK1 as a specific client for Hsp90 and the role of this association in disease.

### 4.1. The Hsp90-PDE6 Chaperone Complex

PDE6, a member of the class I family of phosphodiesterases [60], is a heterotetrametric complex, which in rod photoreceptors, comprises the catalytic PDE6α and PDE6β subunits together with two inhibitory PDE6γ subunits. Cone PDE6 comprises two catalytic PDE6α’ subunits and two inhibitory PDE6γ’ subunits. In the phototransduction cascade, activated transducin relieves the inhibition of the PDE6 catalytic subunits imposed by the inhibitory subunits, leading to cGMP hydrolysis. Mutations in rod PDE6α, PDE6β, and PDE6γ cause autosomal recessive RP and mutations in PDE6β can also cause autosomal dominant congenital stationary night blindness (https://sph.uth.edu/retnet/, accessed on 3 July 2022). In contrast, mutations in cone PDE6α’ and PDE6γ’ are associated with autosomal recessive cone or cone–rod dystrophy, or achromatopsia (https://sph.uth.edu/retnet/, accessed on 3 July 2022). Interestingly, whilst mutations in the PDE6 subunits cause relatively milder forms of inherited retinal degeneration, mutations in the reported co-chaperone for PDE6, the photoreceptor-specific aryl hydrocarbon receptor interacting protein-like 1 (AIPL1), cause Leber congenital amaurosis (LCA), a severe early onset and rapidly progressive disease leading to photoreceptor degeneration and the loss of vision within the first few years of life [61]. An early observation in *Aipl1* knockout and knockdown mice was the post-transcriptional loss of all three subunits of rod PDE6 prior to the onset of retinal degeneration [62,63]. Cone PDE6 levels were also substantially reduced in cone photoreceptors lacking AIPL1 [64]. In the absence of AIPL1, the rod PDE6 subunits were stably synthesized but subsequently misassembled and targeted for rapid proteasomal degradation [65]. Similarly, whilst the loss of AIPL1 had no effect on the synthesis of the cone PDE6 subunits, the translated subunits were unstable and could not assemble into the holoenzyme [66]. These studies confirmed that AIPL1 is important for the post-translational stability and assembly of both rod and cone PDE6.

#### 4.1.1. AIPL1 Structure

**Figure 2:**
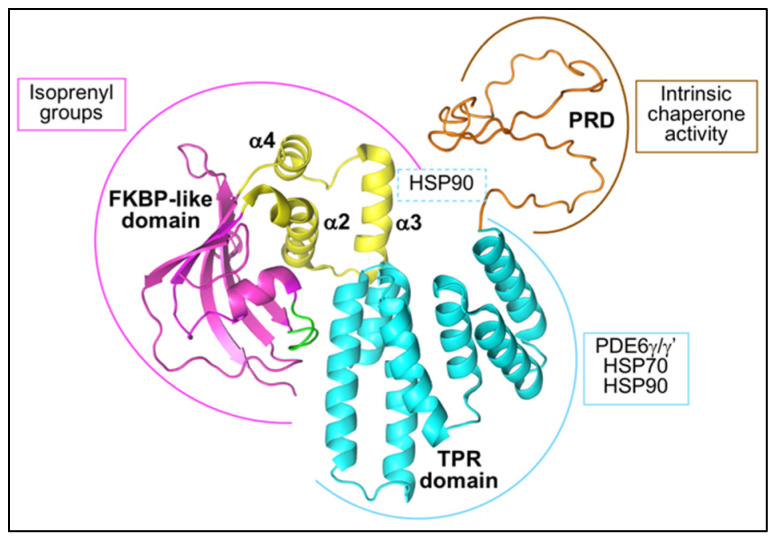
Model of AIPL1. The TPR domain (PDB 6PX0, cyan) and the FKBP-like domain (PDB 5U9A, magenta, yellow, and green) of AIPL1 were superimposed onto FKBP51 (1KT0). The PRD (brown) of AIPL1 was modelled using the I-TASSER server [74,75]. Helices 2, 3, and 4 of the unique insert region of the AIPL1 FKBP51-like domain (yellow) and the loop between β4 and α1 that adopts a ‘looped-out’ conformation in AIPL1 (green) are shown. The PRD is required for the intrinsic chaperone activity of AIPL1. The TPR domain mediates the interaction with Hsp90/Hsp70 and the PDE6 inhibitory subunits, whilst Hsp90 may also make contact with the α3 helix in the unique insert region. The FKBP-like domain constitutes a ligand-binding site for isoprenyl groups. Figure courtesy of C. Prodromou, Genome Damage and Stability Centre, University of Sussex.

AIPL1 was first identified as a possible Hsp90 co-chaperone due to its homology to the Hsp90 tetratricopeptide repeat (TPR) domain co-chaperone aryl hydrocarbon receptor interacting protein (AIP), with which it shares 49% identity and 69% similarity [61]. AIPL1 and AIP comprise a C-terminal TPR domain and a N-terminal FK506 binding protein (FKBP)-like domain, similar to larger members of the FKBP family of immunophilins, including the Hsp90 TPR domain co-chaperones FKBP51 and FKBP52. The AIPL1 TPR domain consists of three consecutive TPR motifs, and the crystal structure of the human AIPL1 TPR domain revealed that, similar to other Hsp90 TPR domain co-chaperones, the AIPL1 TPR domain adopts a typical TPR fold [67] (Figure 2). Each TPR motif consists of a pair of anti-parallel α-helices such that the consecutive TPR motifs form a series of six anti-parallel α-helices connected by short loops followed by a seventh α-helix, which all together forms a right-handed amphipathic groove. The AIPL1 FKBP-like domain shares the typical FKBP fold comprising a five stranded β sheet forming a half β-barrel surrounding a short α helix and creating a hydrophobic cavity (Figure 2). However, unlike other members of the FKBP family, the FKBP-like domain of AIP and AIPL1 lack peptidyl prolyl isomerase activity and cannot bind immunosuppressant drugs [68,69]. Moreover, the FKBP-like domain of both AIP and AIPL1 uniquely include an extensive insert region linking the last two β strands in the FKBP-like domain [69,70,71]. In AIP, the insert regions consist of a 19 residue long helical segment followed by a mostly random coil structure and an α-helix [69]. In contrast, the crystal structure of the human AIPL1 FKBP-like domain revealed that the insert region in AIPL1 (residues 90–146) is well structured and comprises three consecutive α-helices (α2, α3 and α4) connected by short loops [71] (Figure 2). Additional differences between the AIP and AIPL1 FKBP-like domains include the absence of an N-terminal α-helix in AIPL1 that is thought to structurally stabilize the AIP FKBP-like fold; and a loop between β4 and α1 that adopts a ‘looped-out’ conformation in AIPL1 but a ‘looped-in’ conformation in AIP, wherein a critical hinge residue Trp72 is either flipped in or out, respectively, thus modulating access to a hydrophobic cavity [71] (Figure 2). Finally, the AIPL1 TPR domain is followed by a C-terminal 56 amino acid proline rich domain (PRD), an unstructured random coil that is imperfectly conserved in primates and absent in non-primates [61,72,73] (Figure 2).

#### 4.1.2. The Interaction of AIPL1 with Hsp90

Hidalgo-de-Quintana et al. (2008) first provided experimental evidence for the TPR-mediated interaction of full length human AIPL1 with both Hsp90 and Hsp70, with preferential binding to Hsp90 [76]. The TPR consensus residues required for the packing of adjacent α-helices in the TPR motifs and residues involved in tight electrostatic interactions with the C-terminal EEVD TPR acceptor sites of Hsp90 and Hsp70 are conserved in AIPL1. The deletion of the Hsp90 MEEVD pentapeptide or the Hsp70 TIEEVD heptapeptide significantly reduced the interaction of AIPL1 with Hsp90 and Hsp70, respectively, and the MEEVD peptide competitively reduced the interaction of AIPL1 with Hsp90 in quantitative binding assays [76,77]. Moreover, the mutation of lysine 265 to alanine (K265A), a carboxylate clamp residue critical for the tight electrostatic interaction of TPR domain co-chaperones with the C-terminal EEVD motif, significantly reduced the interaction of AIPL1 with Hsp90 and Hsp70 [76,78]. The AIPL1 TPR domain alone can interact with Hsp90 in the absence of the FKBP-like domain, and the disruption of the TPR domain by LCA-associated missense mutations, deletions, insertions, duplications, or C-terminal truncations significantly reduced or abolished the interaction with Hsp90 [77,79]. Therefore, the TPR domain is critical for the interaction of AIPL1 with Hsp90 and features directing the prototypical core TPR domain co-chaperone–chaperone interaction are conserved in AIPL1. Accordingly, Sacristan-Reviriego et al. (2017) showed that human AIPL1 preferentially interacts with Hsp90 in the nucleotide-bound closed conformation and that this interaction is reduced by both apyrase treatment or HSP990 inhibition, indicating that productive Hsp90 ATPase cycles are required for efficient AIPL1 interaction [77]. Moreover, AIPL1 stabilized rod PDE6α to proteasomal degradation in the cytosol and this function was significantly reduced by Hsp90 inhibition with HSP990, GA, or 17-AAG [77]. Similarly, biolayer interferometry (BLI) binding assays recently reported the preferential binding of mouse AIPL1 to adenylyl-imidodiphosphate (AMP-PNP)-bound Hsp90 in a 1:2 stoichiometry [78]. In this study, DMAG treatment significantly impacted the ability of AIPL1 to chaperone cone PDE6α’ activity. Altogether, the data point to the preferential interaction of AIPL1 with Hsp90 in the closed conformation and the importance of a functional AIPL1-Hsp90 interaction for PDE6 stability and activity.

In addition to the role of the core TPR domain contacts in mediating the interaction of AIPL1 with the chaperone TPR acceptor site, additional requirements for this interaction have been investigated. In the case of FKBP51 and FKBP52, a region C-terminal to the TPR domain comprising a seventh α-helical extension (α-helix 7) mediates differential binding to Hsp90 and the truncation of FKBP51 and FKBP52 within this α-helical extension at Asn404 and Asn406, respectively, largely abrogated Hsp90 interaction [80]. Interestingly, the removal of the α-helical extension C-terminal to the core TPR domain of human AIPL1 by truncation at the topologically equivalent residue, Glu317 (AIPL1 1-317), reduced but did not abolish the interaction of AIPL1 with Hsp90 and Hsp70 [76]. Similarly, the truncation of the 12 C-terminal residues of mouse AIPL1 (AIPL1 1-316) did not abrogate the binding of AIPL1 to Hsp90 but moderately reduced the affinity for Hsp90 in BLI assays [78]. Notably, this region was however critical for the ability of AIPL1 to chaperone PDE6 in an in vitro heterologous assay for cone PDE6α’ function. This suggests that in addition to the core TPR domain contacts, residues within the TPR α-helical extension may be important for functional chaperone complex assembly, although several residues thought to mediate contact of the α-helical extension of FKBP51 with Hsp90 are missing or not conserved in mouse or human AIPL1.

Other regions implicated in the interaction of AIPL1 with Hsp90 include the primate-specific PRD and the α3 helix in the unique insert region of the FKBP-like domain. The deletion of the PRD, whilst having no effect on the structure or thermostability of AIPL1 [68,73], was reported to modestly increase the interaction of AIPL1 with Hsp90 in surface plasmon resonance (SPR) spectroscopy assays. On the other hand, the interaction of AIPL1 with Hsp90 following deletion of the PRD was reported to be comparable to that of full length AIPL1, although a significantly increased interaction was observed with the TPR domain alone in the absence of the PRD [79]. Finally, disease-associated mutations in the AIPL1 PRD had no effect on the interaction with Hsp90 [79]. Whilst the PRD may therefore not play a significant role in the interaction with Hsp90, it does appear to play a critical role in the intrinsic chaperone activity of AIPL1 [68]. AIPL1 was first shown to efficiently suppress the formation of intracellular inclusions comprising misfolded fragments of the AIPL1 interacting partner, NUB1, in a concentration-dependent manner [81]. AIPL1 also suppressed the thermal aggregation of citrate synthase (CS) and protected CS from thermal inactivation, and this effect was lost upon the deletion of the PRD [68]. The AIPL1 suppression of aggregation of the NUB1 fragments was not dependent on Hsp90, as GA had no effect in this assay, but was additive with Hsp70 dependent on AIPL1 C-terminal sequences [76]. Overall, the data suggest that the PRD is critical for AIPL1 intrinsic chaperone activity in association with Hsp70.

A number of studies have also investigated the contribution of the AIPL1 FKBP-like domain to Hsp90 interaction. The AIPL1 FKBP-like domain and TPR domain expressed alone can each fold stably to acquire the native conformation [67,70,71,82]. It has been reported that the AIPL1 FKBP-like domain alone, however, cannot interact with Hsp90 in the absence of the TPR domain [77]. Indeed, the LCA-associated patient mutation, Glu163Stop, which leads to the loss of the entire TPR domain and PRD, completely abolished the interaction of AIPL1 with Hsp90, confirming the critical role of the TPR domain in Hsp90 interaction [77]. However, patient-associated mutations in the FKBP-like domain, including missense mutations and in-frame deletions, diminished the interaction of AIPL1 with Hsp90 and impacted rod PDE6 activity in an indirect assay of cGMP hydrolysis [77,79], suggesting that whilst the FKBP-like domain alone cannot bind Hsp90, it is important for stable ternary chaperone complex formation with full length AIPL1. Interestingly, a very weak but transient interaction of Hsp90 with the N-terminal FKBP-like domain of AIP has been reported, and this interaction was reduced by the deletion of the FKBP-like domain unique insert region [69]. Similarly, the replacement of the α3 helix in the AIPL1 FKBP-like unique insert region with five glycine residues modestly affected the interaction with Hsp90, but critically impacted the activity of cone PDE6α’ [78]. A model of the Hsp90-AIPL1 complex based on the cryo-EM structure of the Hsp90-FKBP51 complex placed the α3 helix of the insert region in close proximity to Hsp90, suggesting a moderate contribution of the insert region to the AIPL1–Hsp90 interface [78]. As the TPR acceptor site of Hsp90 can competitively bind a multitude of TPR domain co-chaperones, it has been suggested that contacts with the α3 helix may contribute to the specificity of the interaction of AIPL1 with Hsp90.

#### 4.1.3. The AIPL1-Mediated Targeting of PDE6 to Hsp90

The binding interface between the PDE6 client and Hsp90 has not been investigated. However, several mechanisms have been proposed wherein AIPL1 could specifically target PDE6 to Hsp90. Ramamurthy et al. (2003) first reported that AIPL1 could interact with and facilitate the processing of farnesylated proteins [83]. Notably, the PDE6 catalytic subunits are isoprenylated at the cysteine residue of their C-terminal CAAX box, with the identity of the CAAX box C-terminal residue suggesting that rod PDE6α is farnesylated whilst rod PDE6β and cone PDE6α’ are geranylgeranylated. A general role for AIPL1 in protein farnesylation was suggested, since several interactors in a Y2H screen were farnesylated, and mutation of the CAAX box cysteine to induce the loss of farnesylation or promote geranylgeranylation led to the loss of these interactions [83]. Accordingly, AIPL1 was found to interact with rod PDE6α in the mouse retina, and the AIPL1 interaction with PDE6β was reported to be dependent on that with PDE6α [65]. FRET assays with an AMCA conjugated farnesylated cysteine probe, S-farnesyl-L-cysteine methyl ester, revealed a high affinity interaction with the purified FKBP-like but not the TPR domain [70]. Mutation of Cys89 or Leu147 flanking the unique FBKP insert region or the deletion of the insert region (residues 96-143) abolished the interaction with the probe. Similarly, FRET assays confirmed a potent interaction of AIPL1 with an AMCA-conjugated peptide mimic of the PDE6α C-terminus with the cysteine residue modified by S-farnesylation and carboxymethylation [73]. Interestingly, competition assays with an excess of N-acetyl-S-geranylgeranyl-L-cysteine suggested for the first time that AIPL1 may also bind geranylgeranyl. Indeed, the crystal structure of the AIPL1 FKBP-like domain (residues 2-161) in the apo state and in the presence of either S-farnesyl-L-cysteine methyl ester or geranylgeranyl pyrophosphate confirmed the interaction of AIPL1 with these isoprenoid moieties that bind deep within the hydrophobic cavity [71]. There were no significant differences between the apo and isoprenoid-bound structures, with isoprenoid binding inducing only minor conformational changes in the ligand binding domain. Molecular dynamics simulations supported a model wherein the β4-α1 loop adopts a ‘looped-in’ conformation in the apo structure with the critical Trp72 residue thus occluding the hydrophobic ligand-binding pocket, which then rotates to the ‘flipped-out’ conformation upon isoprenoid binding [71]. The α2 side chains of the insert region were found to contribute significantly to isoprenoid binding [71], explaining the previous observation that the deletion of the insert region abrogated interaction with a farnesyl probe [70]. Moreover, the mutation of residues in the β4-α1 loop also markedly attenuated isoprenoid binding [71]. These studies thus confirmed the direct interaction of the AIPL1 FKBP-like domain with either farnesyl or geranylgeranyl, suggesting that AIPL1 might specifically target PDE6 to Hsp90 through these interactions. Notably, the mutation of the PDE6α’ CAAX-box cysteine to favour either farnesylation or geranylgeranylation had no impact on the ability of AIPL1 to chaperone functional cone PDE6 in in vitro heterologous activity assays, suggesting that the role of AIPL1 is indiscriminate with respect to the identity of the isoprenoid moiety [84]. More recently, a PDE6α Cys857Ser knockin mouse model has been generated that abrogates the farnesylation of rod PDE6α [78]. Interestingly, the levels and targeting of PDE6α and PDE6β to the photoreceptor OS, as well as both the basal and maximal PDE6 activity, were comparable to control mice, in addition to which there was no change in either ERG or optical coherence tomography (OCT) measurements. Moreover, the deletion of the C-terminal 28 residues of cone PDE6α’, including the CAAX motif or the loss of isoprenylation by Cys855Ser mutation, had no effect in in vitro assays of AIPL1 chaperoned PDE6 activity. Finally, steric occlusion of the AIPL1 prenyl binding site in an Ile61Phe/Ile151Phe double mutant had no effect on the ability of AIPL1 to chaperone PDE6α’ or the Cys855Ser mutant in the heterologous assay. Hence, overall, whilst it is clear that the interaction of the AIPL1 FKBP-like domain with the PDE6 isoprenoid moieties contributes to the formation of the ternary chaperone complex, the exact role of this interaction in PDE6 biogenesis remains unclear. It is noteworthy that whilst the FKBP-like domain of AIPL1 appears to bind either farnesyl or geranylgeranyl groups indiscriminately, only PDE6 is affected in the *Aipl1* knockout and knockdown mouse models (in addition to soluble retinal guanylate cyclase in *Aipl1* knockout cones), despite the wide range of phototransduction components that are isoprenylated, thus suggesting that features other than the interaction of AIPL1 with the PDE6 isoprenoid groups must facilitate the specific recruitment of PDE6 to Hsp90 by AIPL1.

One such possibility is the interaction of AIPL1 with the inhibitory subunits of rod and cone PDE6. The rod PDE6γ subunit was reported to interact with AIPL1 using FRET assays [73]. Subsequently, a conserved C-terminal peptide of rod PDE6γ and cone PDE6γ’ was shown to bind the AIPL1 TPR domain but not the AIPL1 FKBP-like domain with association and dissociation kinetics consistent with a 1:1 binding model [67]. Moreover, molecular modelling suggested that the C-terminal 25 residues of the rod and cone inhibitory subunits encompass most, if not all, of the contact with the TPR domain overlapping with the Hsp90 binding site, such that the inhibitory subunits and Hsp90 bind in a mutually exclusive manner [67]. This suggests a model in which the interaction of the PDE6γ/PDE6γ’ subunits with the AIPL1 TPR domain impart specificity toward the PDE6 client, as the AIPL1 FKBP-like domain can bind isoprenyl moieties indiscriminately and the Hsp90 TPR acceptor site is bound competitively by TPR domain co-chaperones. This has important implications for modelling the role of AIPL1 and Hsp90 in PDE6 biogenesis in retinal photoreceptors, though it is noteworthy that AIPL1 failed to interact with either rod PDE6γ or cone PDE6γ’ in co-immunoprecipitation assays of AIPL1 from mouse retinal explants [65,66].

Overall, these studies highlight the structural and mechanistic basis of PDE6 recruitment to Hsp90 via the PDE6-specific Hsp90 co-chaperone AIPL1. Misfolded PDE6 subunits that cause autosomal recessive retinal disease likely undergo unproductive folding cycles with Hsp90, leading to their post-translational degradation and loss of function.

### 4.2. The Hsp90-GRK1 Chaperone Complex

In comparison to the Hsp90-PDE6 chaperone complex, the interaction and maturation of GRK1 with Hsp90 as a client protein in retinal photoreceptors is poorly characterised. Mutations in GRK1 are associated with the Oguchi subtype of recessive congenital stationary night blindness (https://sph.uth.edu/retnet/, accessed on 3 July 2022). In the retina, GRK1 is expressed in rod photoreceptors whilst GRK7 is expressed in cone photoreceptors. Both GRK1 and GRK7 are members of the G protein-coupled receptor kinase (GRK) family, serine/threonine-specific protein kinases that mediate the agonist-dependent phosphorylation of G protein-coupled receptors.

GRK1 and GRK7 specifically target the activated state of the G protein-coupled receptors rhodopsin and cone opsin, respectively, and play a key role in the deactivation of the phototransduction cascade and photorecovery after light onset. GRK1 and GRK7 are tethered to the photoreceptor OS phospholipid membranes in close proximity to their substrate by C-terminal isoprenylation. In the dark, GRK1 and GRK7 are bound to and inhibited by recoverin. The activation of the phototransduction cascade leads to the release of calcium from recoverin, which induces a conformational change involving the rotation of a ‘myristoyl switch’ that results in the calcium-dependent dissociation of recoverin from the membrane and release from GRK, thus enabling GRK to phosphorylate rod and cone opsin [85]. The GRK-mediated phosphorylation of the opsins induces a conformational change in the receptor that in turn allows the binding of arrestin and receptor deactivation through sterically blocking the binding of transducin, effectively switching off the cascade [85].

The GRK family, in addition to the visual kinase subfamily (GRK1, GRK7), includes the β-adrenergic receptor (β-AR) kinase subfamily (GRK2, GRK3) and the GRK4 subfamily (GRK4, GRK5, GRK6). All GRK family members are composed of a short highly conserved ~16 residue N-terminal element unique to this family of kinases [86]. This is followed by a regulator of G protein signalling homology domain (RH) that is interrupted by a highly conserved serine/threonine kinase domain. The catalytic domain of the GRK family, including that of GRK1 and GRK7, has a highly conserved architecture comprising a small N-terminal lobe and a large C-terminal lobe connected by a flexible hinge region that forms a deep nucleotide binding cleft between them, followed by a C-terminal extension (C-tail). The N-terminal lobe comprises a five-stranded β-sheet with a conserved αC helix, whereas the C-terminal lobe comprises six α-helices. A loop within the C-tail forms an active site tether that contributes to the ATP binding site. The substrate mainly interacts with the surface of the C-terminal lobe. The RH domain folds into a bi-lobed helical bundle that bridges the small and large kinase domains [86]. Kinase activation involves a conformational change in which the N-terminal lobe moves towards C-terminal lobe to form a closed state, with the αC-β4 loop thought to act as a hinge point for inter-lobe movement, enabling the rotation between open and closed conformations [87].

Hsp90 has been shown to play a role in the maturation and stabilization of the GRK family members GRK1, GRK2, GRK3, GRK5, and GRK6, with the inhibition of Hsp90 by GA or 17-AAG, leading to rapid proteasomal degradation of the newly synthesized GRKs [43,88]. Similar to GRK1, cone GRK7 is also likely to be an Hsp90 client protein. Hsp90 is known to bind directly to the kinase catalytic domain with Hsp90 binding determinants widely distributed in both lobes. The kinase catalytic domain is one of the most abundant structures in the human proteome, present in more than 500 protein kinases, and has a highly conserved architecture common to serine/threonine and tyrosine kinases. The kinase domain is thus regarded as a universal acceptor site mediating kinase interaction with Hsp90, and it is highly likely, therefore, that the kinase domain of GRK1 and GRK7 similarly directs the interaction with Hsp90. Considerable effort has been invested in identifying the specific features that mediate the recognition of client kinases by Hsp90 [89,90,91,92,93,94]. Interestingly, no global sequence determinants have been identified for the interaction of kinases with Hsp90, despite the high level of conservation in the kinase domain. Instead, a consensus has emerged in which the intrinsic stability of the kinase domain is an important determinant for Hsp90 interaction. Hsp90 kinase clients were reported to be more thermodynamically unstable than non-clients, with the small-molecule stabilization of the kinase domain reducing the client interaction and mutation of the kinase domain, leading to stronger Hsp90 client binding [89,90,93,94].

Finally, it is well known that cell division cycle 37 (Cdc37) is a ubiquitous kinase-dedicated co-chaperone that is universally employed to direct kinase clients to Hsp90, thereby providing selective recognition of the kinase family [93,94]. Cdc37 has been shown to directly bind the kinase catalytic site, overlapping with the Hsp90 binding. Cdc37 binds to kinase clients in the absence of Hsp90, whereas Hsp90 interacts only weakly without Cdc37. Therefore, Hsp90 and Cdc37 are thought to act in concert in chaperoning client kinases with Hsp90-mediated maturation of kinases strictly dependent on the Cdc37-dependent recruitment of the kinase to Hsp90 [93,94]. Whilst not experimentally tested, it is highly likely that the co-chaperone for GRK1 and GRK7 is Cdc37. Experimental investigation of GRK1 and GRK7 interaction with Hsp90 and Cdc73 will provide further insights and evidence that the features directing client kinase assembly with Cdc73 and Hsp90 are conserved amongst the visual GRKs.

## 5. Conclusions

In summary, retinal photoreceptors are amongst the most metabolically active cells in the human body. Consequently, high levels of reactive oxygen species accumulate in the photoreceptors, leading to membrane and protein damage. Approximately 10% of the photoreceptor outer segments are turned over daily to replace damaged membranes and proteins. There is therefore an extremely high demand on protein synthesis and turnover in retinal photoreceptor cells requiring high levels of proteostasis. There are currently 280 genes associated with inherited retinal disease, many of which code for proteins of the visual cycle and phototransduction cascade that require high levels of protein synthesis in the photoreceptor inner segment and translocation to the outer segment. Protein quality control is therefore of vital importance in photoreceptor cells and many inherited retinal diseases are protein misfolding disorders. Hsp90 is centrally important to protein folding and quality control in the retinal photoreceptors, including in the cytosol, ER, and in the mitochondria. Indeed, the induction of the HSR via short-term Hsp90 inhibition has been shown to be neuroprotective in in vitro and in vivo models of inherited retinal disease. However, longer term inhibition of Hsp90 in the retina may in fact be detrimental due to the resultant degradation of specific Hsp90 client proteins in the photoreceptors, including PDE6 and GRK1. Mutations in these Hsp90 client proteins themselves lead to retinal disease. Whilst the precise role of Hsp90 in the folding, maturation, or assembly of these retina-specific client proteins is not fully elucidated, this raises the possibility that small molecule manipulation of the Hsp90 cycle to promote the favourable maturation of these client proteins may be a potential therapeutic approach for diseases associated with these clients. In addition, the induction of the HSR in the absence of Hsp90 inhibition might be another favourable avenue for treating protein misfolding disorders in the retina.

## Figures and Tables

**Table 1:** List of Hsp90 inhibitors used in models of retinal degeneration.

Compound	Study Outcome	Reference
Geldanamycin	Reduced P23H aggregation and cell death in vitro	Mendes & Cheetham, 2008 [42]
Tanepsimycin 17-AAG	Reduced P23H aggregation and cell death in vitro	Mendes & Cheetham, 2008 [42]
	Reduced protein accumulation in R135L rats	Aguilà et al., 2014 [43]
Radicicol	Reduced P23H aggregation and cell death in vitro	Mendes & Cheetham, 2008 [42]
Alvespimycin 17-DMAG	Prolonged treatment causes photoreceptor cell death in rats	Zhou et al., 2013 [46]
	Induced photoreceptor apoptosis and rhodopsin retention in the IS in wild-type mice	Wu et al., 2020 [19]
HSP990	Reduces P23H aggregation, improves visual function and delays photoreceptor cell death in P23H-1 rats	Aguilà et al., 2014 [43]
	Protects photoreceptors from degeneration caused by aggregating mutant IMPDH1 protein	Tam et al., 2010 [45]

**Table 2:** List of Hsp90 inhibitors used in clinical trials in oncology and their ocular effects. * The observed ocular effects were transient or resolved after treatment discontinuation.

HSP90 Inhibitor Drug	Trial	ClinicalTrials.gov Identifier	Ocular Effect	Reference
Alvespimycin 17-dimethylaminoethylamino-17-demethoxygeldanamycin (17-DMAG)	Phase I trial of 17-DMAG in patients with advanced malignancies	NCT00088868	blurred vision *	Kummar et al., 2010 [47]
	Phase I trial of 17-DMAG in patients with advanced solid tumors	NCT00248521	blurred vision dry eye keratitis conjunctivitis or ocular surface disease	Pacey et al., 2011 [50]
Onalespid AT13387	Phase I trial of AT13387 in patients with refractory solid tumors.	NCT00878423	blurred vision * flashes * delayed light dark/accommodation *	Shapiro et al., 2010 [49]
	Phase I study of onalespib in combination with AT7519, a pan-CDK inhibitor, in patients with advanced solid tumors	NCT02503709	blurry vision and “floaters” *	Do et al., 2020 [56]
Luminespid AUY922 NVP-AUY922	Phase I trial of AUY922 in combination with capecitabine in patients with advanced solid tumors	NCT01226732	vision darkening * night blindness *	Bendell et al., 2015 [52]
	Phase I-IB/II trial of NVP-AUY922 as monotherapy or in combination with bortezomib in patients with relapsed or refractory multiple myeloma	NCT00708292	night blindness photopsia visual impairment retinopathy blurred vision cataract reduced visual acuity	Seggewiss-Bernhardt et al., 2015 [53]
	Phase II trial of AUY922 in patients with refractory gastrointestinal stromal tumors	NCT01404650	blurred vision * flashing lights * delayed light/dark adaptation * night blindness * floaters *	Bendell et al., 2016 [54]
	Phase II trial of AUY922 in patients with metastatic gastrointestinal stromal tumor	NCT01389583	night blindness blurred vision flashing light	Chiang et al., 2016 [55] Shen et al., 2021 [57]
SNX-5422 PF-04929113	Phase I study of SNX-5422 in patients with refractory solid tumor malignancies and lymphomas	NCT00644072	blurred vision bilateral cataracts	Rajan et al., 2011 [51]

## Data Availability

Not applicable.

## Abbreviations

17-DMAG, 17-Dimethylaminoethylamino-17-demethoxygeldanamycin; 17-AAG, 17-*N*-allylamino-17-demethoxygeldanamycin; ATF6, activating transcription factor 6; AMP-PNP, adenylyl-imidodiphosphate; AIP, aryl hydrocarbon receptor interacting protein; AIPL1, aryl hydrocarbon receptor interacting protein-like 1; adRP, autosomal dominant retinitis pigmentosa; Cdc37, cell division cycle 37; CS, citrate synthase; CC, connecting cilium; ER, Endoplasmic reticulum; ERAD, ER-associated degradation; FKBP, FK506 binding protein; GRK1, G-protein-coupled receptor kinase 1; GA, geldanamycin; GRP94, glucose-regulated protein 94; GC, guanylate cyclase; GCAP, guanylyl cyclase activating proteins; HSE, heat shock element; HSF, heat shock factor; HSP, heat shock protein; HSR, heat shock response; HSP990, 2-amino-7,8-dihydro-6H-pyrido[4,3-D]pyrimidin-5-one NVPHSP990; IS, inner segment; IMPDH1, inosine 5′-monophosphate dehydrogenase type 1; IRE1, inositol-requiring protein-1; LCA, Leber congenital amaurosis; MAP1B, microtubule-associated protein 1B; UPR^mt^, mitochondrial unfolded protein response; OCT, optical coherence tomography; OS, outer segment; PRD, proline rich domain; PERK, protein kinase RNA (PKR)-like ER kinase; PQC, protein quality control; PDE6, retinal phosphodiesterase; RPE, retinal pigment epithelium; RP, Retinitis pigmentosa; RGS9, regulator of G-protein-signalling isoform 9; SPR, surface plasmon resonance; TPR, tetratricopeptide repeat; TRAP1, tumor necrosis factor receptor associated protein 1; UPR, unfolded protein response.

## References

[1] Strauss O. (2005). The Retinal Pigment Epithelium in Visual Function. Physiol. Rev..

[2] Athanasiou D., Aguila M., Bellingham J., Li W., McCulley C., Reeves P.J., Cheetham M.E. (2018). The molecular and cellular basis of rhodopsin retinitis pigmentosa reveals potential strategies for therapy. Prog. Retin. Eye Res..

[3] Arshavsky V.Y., Lamb T.D., Pugh E.N. (2003). G Proteins and Phototransduction. Annu. Rev. Physiol..

[4] Arshavsky V.Y., Wensel T.G. (2013). Timing Is Everything: GTPase Regulation in Phototransduction. Investig. Opthalmology Vis. Sci..

[5] Kefalov V. (2010). Phototransduction: Phototransduction in Cones. Encycl. Eye.

[6] Ingram N.T., Sampath A.P., Fain G.L. (2016). Why are rods more sensitive than cones?. J. Physiol..

[7] Spencer W.J., Lewis T., Pearring J.N., Arshavsky V.Y. (2020). Photoreceptor Discs: Built Like Ectosomes. Trends Cell Biol..

[8] Léveillard T., Sahel J.-A. (2017). Metabolic and redox signaling in the retina. Cellular and Molecular Life Sciences.

[9] Narayan D.S., Chidlow G., Wood J.P.M., Casson R.J. (2017). Glucose metabolism in mammalian photoreceptor inner and outer segments. Clin. Exp. Ophthalmol..

[10] Kampinga H.H., Hageman J., Vos M.J., Kubota H., Tanguay R.M., Bruford E.A., Cheetham M.E., Chen B., Hightower L.E. (2009). Guidelines for the nomenclature of the human heat shock proteins. Cell Stress Chaperones.

[11] Biebl M.M., Buchner J. (2019). Structure, Function, and Regulation of the Hsp90 Machinery. Cold Spring Harb. Perspect. Biol..

[12] Prodromou C., Bjorklund D.M. (2022). Advances towards Understanding the Mechanism of Action of the Hsp90 Complex. Biomolecules.

[13] Åkerfelt M., Morimoto R.I., Sistonen L. (2010). Heat shock factors: Integrators of cell stress, development and lifespan. Nat. Rev. Mol. Cell Biol..

[14] Voellmy R. (2004). On mechanisms that control heat shock transcription factor activity in metazoan cells. Cell Stress Chaperon..

[15] Gomez-Pastor R., Burchfiel E.T., Thiele D.J. (2017). Regulation of heat shock transcription factors and their roles in physiology and disease. Nat. Rev. Mol. Cell Biol..

[16] Prodromou C. (2016). Mechanisms of Hsp90 regulation. Biochem. J..

[17] Luengo T.M., Kityk R., Mayer M.P., Rüdiger S.G. (2018). Hsp90 Breaks the Deadlock of the Hsp70 Chaperone System. Mol. Cell.

[18] Hoter A., El-Sabban M.E., Naim H.Y. (2018). The HSP90 Family: Structure, Regulation, Function, and Implications in Health and Disease. Int. J. Mol. Sci..

[19] Wu Y., Zheng X., Ding Y., Zhou M., Wei Z., Liu T., Liao K. (2020). The molecular chaperone Hsp90α deficiency causes retinal degeneration by disrupting Golgi organization and vesicle transportation in photoreceptors. J. Mol. Cell Biol..

[20] Marzec M., Eletto D., Argon Y. (2012). GRP94: An HSP90-like protein specialized for protein folding and quality control in the endoplasmic reticulum. Biochim. et Biophys. Acta-Mol. Cell Res..

[21] Meunier L., Usherwood Y.-K., Chung K.T., Hendershot L.M. (2002). A Subset of Chaperones and Folding Enzymes Form Multiprotein Complexes in Endoplasmic Reticulum to Bind Nascent Proteins. Mol. Biol. Cell.

[22] Hetz C., Zhang K., Kaufman R.J. (2020). Mechanisms, regulation and functions of the unfolded protein response. Nat. Rev. Mol. Cell Biol..

[23] Anukanth A., Khorana H. (1994). Structure and function in rhodopsin. Requirements of a specific structure for the intradiscal domain. J. Biol. Chem..

[24] Christianson J.C., Shaler T.A., Tyler R.E., Kopito R.R. (2008). OS-9 and GRP94 deliver mutant α1-antitrypsin to the Hrd1–SEL1L ubiquitin ligase complex for ERAD. Nat. Cell Biol..

[25] Kroeger H., Messah C., Ahern K., Gee J., Joseph V., Matthes M.T., Yasumura D., Gorbatyuk M.S., Chiang W.-C., LaVail M.M. (2012). Induction of Endoplasmic Reticulum Stress Genes, *BiP* and *Chop*, in Genetic and Environmental Models of Retinal Degeneration. Investig. Opthalmology Vis. Sci..

[26] Saliba R.S., Munro P.M.G., Luthert P.J., Cheetham M.E. (2002). The cellular fate of mutant rhodopsin: Quality control, degradation and aggresome formation. J. Cell Sci..

[27] Carreras-Sureda A., Pihán P., Hetz C. (2017). Calcium signaling at the endoplasmic reticulum: Fine-tuning stress responses. Cell Calcium.

[28] Di Jeso B., Ulianich L., Pacifico F., Leonardi A., Vito P., Consiglio E., Formisano S., Arvan P. (2003). Folding of thyroglobulin in the calnexin/calreticulin pathway and its alteration by loss of Ca2+ from the endoplasmic reticulum. Biochem. J..

[29] Mekahli D., Bultynck G., Parys J., De Smedt H., Missiaen L. (2011). Endoplasmic-Reticulum Calcium Depletion and Disease. Cold Spring Harb. Perspect. Biol..

[30] Preissler S., Rato C., Yan Y., Perera L.A., Czako A., Ron D. (2020). Calcium depletion challenges endoplasmic reticulum proteostasis by destabilising BiP-substrate complexes. eLife.

[31] Wengert L.A., Backe S.J., Bourboulia D., Mollapour M., Woodford M.R. (2022). TRAP1 Chaperones the Metabolic Switch in Cancer. Biomolecules.

[32] Felts S.J., Owen B.A.L., Nguyen P., Trepel J., Donner D.B., Toft D.O. (2000). The hsp90-related Protein TRAP1 Is a Mitochondrial Protein with Distinct Functional Properties. J. Biol. Chem..

[33] Haynes C.M., Ron D. (2010). The mitochondrial UPR—Protecting organelle protein homeostasis. J. Cell Sci..

[34] Martinus R.D., Garth G.P., Webster T.L., Cartwright P., Naylor D.J., Høj P.B., Hoogenraad N.J. (1996). Selective Induction of Mitochondrial Chaperones in Response to Loss of the Mitochondrial Genome. Eur. J. Biochem..

[35] Zhao Q., Wang J., Levichkin I.V., Stasinopoulos S., Ryan M., Hoogenraad N.J. (2002). A mitochondrial specific stress response in mammalian cells. EMBO J..

[36] Houtkooper R., Mouchiroud L., Ryu D., Moullan N., Katsyuba E., Knott G.W., Williams R.W., Auwerx J. (2013). Mitonuclear protein imbalance as a conserved longevity mechanism. Nature.

[37] Münch C. (2018). The different axes of the mammalian mitochondrial unfolded protein response. BMC Biol..

[38] Baqri R.M., Pietron A.V., Gokhale R.H., Turner B.A., Kaguni L.S., Shingleton A.W., Kunes S., Miller K.E. (2014). Mitochondrial chaperone TRAP1 activates the mitochondrial UPR and extends healthspan in Drosophila. Mech. Ageing Dev..

[39] Takemoto K., Miyata S., Takamura H., Katayama T., Tohyama M. (2011). Mitochondrial TRAP1 regulates the unfolded protein response in the endoplasmic reticulum. Neurochem. Int..

[40] Georgiou M., Fujinami K., Michaelides M. (2021). Inherited retinal diseases: Therapeutics, clinical trials and end points—A review. Clin. Exp. Ophthalmol..

[41] Parfitt D.A., Aguila M., McCulley C.H., Bevilacqua D., Mendes H.F., Athanasiou D., Novoselov S.S., Kanuga N., Munro P.M., Coffey P.J. (2014). The heat-shock response co-inducer arimoclomol protects against retinal degeneration in rhodopsin retinitis pigmentosa. Cell Death Dis..

[42] Mendes H.F., Cheetham M.E. (2008). Pharmacological manipulation of gain-of-function and dominant-negative mechanisms in rhodopsin retinitis pigmentosa. Hum. Mol. Genet..

[43] Aguilà M., Bevilacqua D., McCulley C., Schwarz N., Athanasiou D., Kanuga N., Novoselov S.S., Lange C.A., Ali R.R., Bainbridge J.W. (2014). Hsp90 inhibition protects against inherited retinal degeneration. Hum. Mol. Genet..

[44] Kennan A., Aherne A., Palfi A., Humphries M., McKee A., Stitt A., Simpson D.A.C., Demtroder K., Orntoft T., Ayuso C. (2002). Identification of an IMPDH1 mutation in autosomal dominant retinitis pigmentosa (RP10) revealed following comparative microarray analysis of transcripts derived from retinas of wild-type and Rho-/- mice. Hum. Mol. Genet..

[45] Tam L.C., Kiang A.-S., Campbell M., Keaney J., Farrar G.J., Humphries M.M., Kenna P.F., Humphries P. (2010). Prevention of autosomal dominant retinitis pigmentosa by systemic drug therapy targeting heat shock protein 90 (Hsp90). Hum. Mol. Genet..

[46] Zhou D., Liu Y., Ye J., Ying W., Ogawa L.S., Inoue T., Tatsuta N., Wada Y., Koya K., Huang Q. (2013). A rat retinal damage model predicts for potential clinical visual disturbances induced by Hsp90 inhibitors. Toxicol. Appl. Pharmacol..

[47] Kummar S., Gutierrez M.E., Gardner E.R., Chen X., Figg W.D., Zajac-Kaye M., Chen M., Steinberg S.M., Muir C.A., Yancey M.A. (2010). Phase I trial of 17-dimethylaminoethylamino-17-demethoxygeldanamycin (17-DMAG), a heat shock protein inhibitor, administered twice weekly in patients with advanced malignancies. Eur. J. Cancer.

[48] Samuel T.A., Sessa C., Britten C., Milligan K.S., Mita M.M., Banerji U., Pluard T.J., Stiegler P., Quadt C., Shapiro G. (2010). AUY922, a novel HSP90 inhibitor: Final results of a first-in-human study in patients with advanced solid malignancies. J. Clin. Oncol..

[49] Shapiro G., Kwak E.L., Dezube B.J., Lawrence D.P., Cleary J.M., Lewis S., Squires M., Lock V., Lyons J.F., Yule M. (2010). Phase I pharmacokinetic and pharmacodynamic study of the heat shock protein 90 inhibitor AT13387 in patients with refractory solid tumors. J. Clin. Oncol..

[50] Pacey S., Wilson R.H., Walton M., Eatock M.M., Hardcastle A., Zetterlund A., Arkenau H.-T., Moreno-Farre J., Banerji U., Roels B. (2011). A Phase I Study of the Heat Shock Protein 90 Inhibitor Alvespimycin (17-DMAG) Given Intravenously to Patients with Advanced Solid Tumors. Clin. Cancer Res..

[51] Rajan A., Kelly R.J., Trepel J.B., Kim Y.S., Alarcon S.V., Kummar S., Gutierrez M., Crandon S., Zein W.M., Jain L. (2011). A phase I study of PF-04929113 (SNX-5422), an orally bioavailable heat shock protein 90 inhibitor, in patients with refractory solid tumor malignancies and lymphomas. Clin. Cancer Res..

[52] Bendell J.C., Jones S.F., Hart L., Pant S., Moyhuddin A., Lane C.M., Earwood C., Murphy P., Patton J., Penley W.C. (2015). A Phase I Study of the Hsp90 Inhibitor AUY922 plus Capecitabine for the Treatment of Patients with Advanced Solid Tumors. Cancer Investig..

[53] Seggewiss-Bernhardt R., Bargou R.C., Goh Y.T., Stewart A.K., Spencer A., Alegre A., Bladé J., Ottmann O.G., Fernandez-Ibarra C., Lu H. (2015). Phase 1/1B trial of the heat shock protein 90 inhibitor NVP-AUY922 as monotherapy or in combination with bortezomib in patients with relapsed or refractory multiple myeloma. Cancer.

[54] Bendell J.C., Bauer T.M., Lamar R., Joseph M., Penley W., Thompson D.S., Spigel D.R., Owera R., Lane C.M., Earwood C. (2016). A Phase 2 Study of the Hsp90 Inhibitor AUY922 as Treatment for Patients with Refractory Gastrointestinal Stromal Tumors. Cancer Investig..

[55] Chiang N.-J., Yeh K.-H., Chiu C.-F., Chen J.-S., Yen C.-C., Lee K.-D., Lin Y.-L., Bai L.-Y., Chen M.-H., Lin J.-S. (2016). Results of Phase II trial of AUY922, a novel heat shock protein inhibitor in patients with metastatic gastrointestinal stromal tumor (GIST) and imatinib and sunitinib therapy. J. Clin. Oncol..

[56] Do K.T., Coyne G.O., Hays J.L., Supko J.G., Liu S.V., Beebe K., Neckers L., Trepel J.B., Lee M.-J., Smyth T. (2020). Phase 1 study of the HSP90 inhibitor onalespib in combination with AT7519, a pan-CDK inhibitor, in patients with advanced solid tumors HHS Public Access. Cancer Chemother. Pharmacol..

[57] Shen C.H., Hsieh C.C., Jiang K.Y., Lin C.Y., Chiang N.J., Li T.W., Yen C.T., Chen W.J., Hwang D.Y., Chen L.T. (2021). AUY922 induces retinal toxicity through attenuating TRPM1. J. Biomed. Sci..

[58] Kanamaru C., Yamada Y., Hayashi S., Matsushita T., Suda A., Nagayasu M., Kimura K., Chiba S. (2014). Retinal toxicity induced by small-molecule Hsp90 inhibitors in beagle dogs. J. Toxicol. Sci..

[59] Roman D., VerHoeve J., Schadt H., Vicart A., Walker U.J., Turner O., Richardson T.A., Wolford S.T., Miller P.E., Zhou W. (2016). Ocular toxicity of AUY922 in pigmented and albino rats. Toxicol. Appl. Pharmacol..

[60] Cote R.H. (2021). Photoreceptor phosphodiesterase (PDE6): Activation and inactivation mechanisms during visual transduction in rods and cones. Pflug. Arch. Eur. J. Physiol..

[61] Sohocki M.M., Bowne S.J., Sullivan L.S., Blackshaw S., Cepko C.L., Payne A., Bhattacharya S.S., Khaliq S., Mehdi S.Q., Birch D. (2000). Mutations in a new photoreceptor-pineal gene on 17p cause Leber congenital amaurosis. Nat. Genet..

[62] Liu X., Bulgakov O.V., Wen X.H., Woodruff M.L., Pawlyk B., Yang J., Fain G.L., Sandberg M.A., Makino C.L., Li T. (2004). AIPL1, the protein that is defective in Leber congenital amaurosis, is essential for the biosynthesis of retinal rod cGMP phosphodiesterase. Proc. Natl. Acad. Sci. USA.

[63] Ramamurthy V., Niemi G.A., Reh T.A., Hurley J.B. (2004). Leber congenital amaurosis linked to AIPL1: A mouse model reveals destabilization of cGMP phosphodiesterase. Proc. Natl. Acad. Sci. USA.

[64] Kirschman L.T., Kolandaivelu S., Frederick J.M., Dang L., Goldberg A.F., Baehr W., Ramamurthy V. (2009). The Leber congenital amaurosis protein, AIPL1, is needed for the viability and functioning of cone photoreceptor cells. Hum. Mol. Genet..

[65] Kolandaivelu S., Huang J., Hurley J.B., Ramamurthy V. (2009). AIPL1, a Protein Associated with Childhood Blindness, Interacts with α-Subunit of Rod Phosphodiesterase (PDE6) and Is Essential for Its Proper Assembly. J. Biol. Chem..

[66] Kolandaivelu S., Singh R.K., Ramamurthy V. (2013). AIPL1, A protein linked to blindness, is essential for the stability of enzymes mediating cGMP metabolism in cone photoreceptor cells. Hum. Mol. Genet..

[67] Yadav R.P., Boyd K., Yu L., Artemyev N.O. (2019). Interaction of the tetratricopeptide repeat domain of aryl hydrocarbon receptor–interacting protein–like 1 with the regulatory Pγ subunit of phosphodiesterase 6. J. Biol. Chem..

[68] Li J., Zoldak G., Kriehuber T., Soroka J., Schmid F.X., Richter K., Buchner J. (2013). Unique Proline-Rich Domain Regulates the Chaperone Function of AIPL1. Biochemistry.

[69] Linnert M., Lin Y.-J., Manns A., Haupt K., Paschke A.-K., Fischer G., Weiwad M., Lücke C. (2013). The FKBP-Type Domain of the Human Aryl Hydrocarbon Receptor-Interacting Protein Reveals an Unusual Hsp90 Interaction. Biochemistry.

[70] Majumder A., Gopalakrishna K.N., Cheguru P., Gakhar L., Artemyev N.O. (2013). Interaction of Aryl Hydrocarbon Receptor-interacting Protein-like 1 with the Farnesyl Moiety. J. Biol. Chem..

[71] Yadav R.P., Gakhar L., Yu L., Artemyev N.O. (2017). Unique structural features of the AIPL1–FKBP domain that support prenyl lipid binding and underlie protein malfunction in blindness. Proc. Natl. Acad. Sci. USA.

[72] Sohocki M.M., Sullivan L.S., Tirpak D.L., Daiger S.P. (2001). Comparative analysis of aryl-hydrocarbon receptor interacting protein-like 1 (Aipl1), a gene associated with inherited retinal disease in humans. Mamm. Genome.

[73] Yadav R.P., Majumder A., Gakhar L., Artemyev N.O. (2015). Extended conformation of the proline-rich domain of human aryl hydrocarbon receptor-interacting protein-like 1: Implications for retina disease. J. Neurochem..

[74] Roy A., Kucukural A., Zhang Y. (2010). I-TASSER: A unified platform for automated protein structure and function prediction. Nat. Protoc..

[75] Zhang Y. (2008). I-TASSER server for protein 3D structure prediction. BMC Bioinform..

[76] Hidalgo-De-Quintana J., Evans R.J., Cheetham M., Van Der Spuy J. (2008). The Leber Congenital Amaurosis Protein AIPL1 Functions as Part of a Chaperone Heterocomplex. Investig. Opthalmology Vis. Sci..

[77] Sacristan-Reviriego A., Bellingham J., Prodromou C., Boehm A.N., Aichem A., Kumaran N., Bainbridge J., Michaelides M., Van Der Spuy J. (2017). The integrity and organization of the human AIPL1 functional domains is critical for its role as a HSP90-dependent co-chaperone for rod PDE6. Hum. Mol. Genet..

[78] Yadav R.P., Boyd K., Artemyev N.O. (2022). Molecular insights into the maturation of phosphodiesterase 6 by the specialized chaperone complex of HSP90 with AIPL1. J. Biol. Chem..

[79] Sacristan-Reviriego A., Le H.M., Georgiou M., Meunier I., Bocquet B., Roux A.-F., Prodromou C., Bainbridge J., Michaelides M., van der Spuy J. (2020). Clinical and functional analyses of AIPL1 variants reveal mechanisms of pathogenicity linked to different forms of retinal degeneration. Sci. Rep..

[80] Cheung-Flynn J., Roberts P.J., Riggs D.L., Smith D.F. (2003). C-terminal Sequences outside the Tetratricopeptide Repeat Domain of FKBP51 and FKBP52 Cause Differential Binding to Hsp90. J. Biol. Chem..

[81] van der Spuy J., Cheetham M. (2004). The Leber Congenital Amaurosis Protein AIPL1 Modulates the Nuclear Translocation of NUB1 and Suppresses Inclusion Formation by NUB1 Fragments. J. Biol. Chem..

[82] Yu L., Yadav R.P., Artemyev N.O. (2017). NMR resonance assignments of the FKBP domain of human aryl hydrocarbon receptor-interacting protein-like 1 (AIPL1) in complex with a farnesyl ligand. Biomol. NMR Assign..

[83] Ramamurthy V., Roberts M., Van den Akker F., Niemi G., Reh T.A., Hurley J.B. (2003). AIPL1, a protein implicated in Leber’s congenital amaurosis, interacts with and aids in processing of farnesylated proteins. Proc. Natl. Acad. Sci. USA.

[84] Gopalakrishna K.N., Boyd K., Yadav R.P., Artemyev N.O. (2016). Aryl Hydrocarbon Receptor-interacting Protein-like 1 Is an Obligate Chaperone of Phosphodiesterase 6 and Is Assisted by the γ-Subunit of Its Client. J. Biol. Chem..

[85] Zang J., Neuhauss S.C.F. (2018). The Binding Properties and Physiological Functions of Recoverin. Front. Mol. Neurosci..

[86] Chen Q., Plasencia M., Li Z., Mukherjee S., Patra D., Chen C.-L., Klose T., Yao X.-Q., Kossiakoff A.A., Chang L. (2021). Structures of rhodopsin in complex with G-protein-coupled receptor kinase 1. Nature.

[87] Yeung W., Ruan Z., Kannan N. (2020). Emerging roles of the αC-β4 loop in protein kinase structure, function, evolution, and disease. IUBMB Life.

[88] Luo J., Benovic J.L. (2003). G Protein-coupled Receptor Kinase Interaction with Hsp90 Mediates Kinase Maturation. J. Biol. Chem..

[89] Xu W., Yuan X., Xiang Z., Mimnaugh E., Marcu M., Neckers L. (2005). Surface charge and hydrophobicity determine ErbB2 binding to the Hsp90 chaperone complex. Nat. Struct. Mol. Biol..

[90] Citri A., Harari D., Shohat G., Ramakrishnan P., Gan J., Lavi S., Eisenstein M., Kimchi A., Wallach D., Pietrokovski S. (2006). Hsp90 Recognizes a Common Surface on Client Kinases. J. Biol. Chem..

[91] Caplan A.J., Mandal A.K., Theodoraki M. (2007). Molecular chaperones and protein kinase quality control. Trends Cell Biol..

[92] Taipale M., Jarosz D.F., Lindquist S. (2010). HSP90 at the hub of protein homeostasis: Emerging mechanistic insights. Nat. Rev. Mol. Cell Biol..

[93] Taipale M., Krykbaeva I., Koeva M., Kayatekin C., Westover K.D., Karras G.I., Lindquist S. (2012). Quantitative Analysis of Hsp90-Client Interactions Reveals Principles of Substrate Recognition. Cell.

[94] Verba K.A., Agard D.A. (2017). How Hsp90 and Cdc37 Lubricate Kinase Molecular Switches. Trends Biochem. Sci..

